# A systematic review of suicide prevention interventions targeting indigenous peoples in Australia, United States, Canada and New Zealand

**DOI:** 10.1186/1471-2458-13-463

**Published:** 2013-05-13

**Authors:** Anton C Clifford, Christopher M Doran, Komla Tsey

**Affiliations:** 1 Institute for Urban Indigenous Health, Edgar Street, Bowen Hills, QLD, 4006, Australia; 2 Hunter Medical Research Centre, University of Newcastle, HMRI Building, Kookaburra Circuit, New Lambton Heights, NSW, 2305, Australia; 3 Education for Social Sustainability, School of Education, Cairns Institute, James Cook University, McGregor Rd, Smithfield, QLD, 4878, Australia

## Abstract

**Background:**

Indigenous peoples of Australia, Canada, United States and New Zealand experience disproportionately high rates of suicide. As such, the methodological quality of evaluations of suicide prevention interventions targeting these Indigenous populations should be rigorously examined, in order to determine the extent to which they are effective for reducing rates of Indigenous suicide and suicidal behaviours. This systematic review aims to: 1) identify published evaluations of suicide prevention interventions targeting Indigenous peoples in Australia, Canada, United States and New Zealand; 2) critique their methodological quality; and 3) describe their main characteristics.

**Methods:**

A systematic search of 17 electronic databases and 13 websites for the period 1981–2012 (inclusive) was undertaken. The reference lists of reviews of suicide prevention interventions were hand-searched for additional relevant studies not identified by the electronic and web search. The methodological quality of evaluations of suicide prevention interventions was assessed using a standardised assessment tool.

**Results:**

Nine evaluations of suicide prevention interventions were identified: five targeting Native Americans; three targeting Aboriginal Australians; and one First Nation Canadians. The main intervention strategies employed included: Community Prevention, Gatekeeper Training, and Education. Only three of the nine evaluations measured changes in rates of suicide or suicidal behaviour, all of which reported significant improvements. The methodological quality of evaluations was variable. Particular problems included weak study designs, reliance on self-report measures, highly variable consent and follow-up rates, and the absence of economic or cost analyses.

**Conclusions:**

There is an urgent need for an increase in the number of evaluations of preventive interventions targeting reductions in Indigenous suicide using methodologically rigorous study designs across geographically and culturally diverse Indigenous populations. Combining and tailoring best evidence and culturally-specific individual strategies into one coherent suicide prevention program for delivery to whole Indigenous communities and/or population groups at high risk of suicide offers considerable promise.

## Background

Indigenous peoples of Australia, Canada, United States (US) and New Zealand have rates of suicide two to three times higher than their country's general population [1-5]. In Australia, suicide accounted for approximately 4% of all deaths in the Aboriginal & Torres Strait Islander population in 2010, versus 1.6% of all deaths in the general Australian population for the same period [1]. The rate of suicide among Canadian First Nation people is at least two times that of Canada's general population [3]. In the United States (US), the rate of suicide among the American Indian population is approximately 1.5 times that of the general US population [4]. In New Zealand, a significant increase in the Maori suicide rate has been observed, particularly among young males [5,6].

National data on Indigenous suicide conceals significant variability in rates and patterns of suicide deaths between regions and communities [7,8]. For example, epidemiological studies have found significant differences in rates of Aboriginal youth suicide between Indigenous tribal councils located in the same Canadian province [7], and clusters [9] of suicide deaths in discrete remote Aboriginal communities in Australia [10,11] and American Indian reservations in the US [12]. Suicide rates in Indigenous populations are also disproportionately higher among younger, relative to older, people, and among non-Indigenous people of the same age [8]. For instance, in Australia, almost half of the health gap between Aboriginal & Torres Strait Islander Australians and other Australians due to injury is attributable to suicide in young Aboriginal males [13], and in New Zealand, suicide rates in Māori youth are more than double that of non- Māori youth [6].

The main risk factors for suicide are mental health disorders, stressful life events and substance abuse [14,15]. All of these risk factors occur at disproportionately high rates in Indigenous populations, placing them at significantly higher risk of suicide than the general population [16,17]. For instance, Indigenous peoples are more likely than the general population to use alcohol and some drugs at levels that increase their risk of mental health disorders [18], and their higher levels of social disadvantage increases their exposure to stressful life events, such as unemployment, homelessness, incarceration and family problems [19], that, in turn, have been shown to increase ones risk of suicide [20]. Indigenous peoples of Australia, New Zealand, Canada and the United States are also at an increased risk of suicidal behaviour due to factors embedded in their historical experiences, including loss of land and culture, trans-generational trauma, grief and loss, racism and social exclusion [18-23]. Indigenous peoples’ continued exposure to multiple risk factors for suicide underscores their urgent need for suicide prevention interventions.

There is evidence from systematic reviews for the effectiveness of different suicide prevention interventions [14,24,25]. This evidence, however, largely derives from evaluations of suicide prevention interventions targeting the general population. Although there are published reviews of suicide interventions specifically targeting Indigenous populations [26-29], a systematic review of published evaluations of suicide prevention interventions targeting Indigenous populations is timely for at least two reasons. Firstly, with the exception of one review examining approaches for reducing suicide among Indigenous youth [29], there have been no published systematic reviews of suicide preventive interventions targeting Indigenous peoples of Australia, New Zealand, Canada and the United States. Outcomes of suicide prevention interventions targeting an Indigenous population in one of these countries may be applicable to Indigenous populations in the other countries, in so far as they exist as formerly colonized peoples that receive a significant portion of health and social services from members and institutions of their settler colonial society. [8,16] Secondly, existing systematic reviews of suicide prevention interventions targeting Indigenous peoples focus on describing the interventions, rather than examining the methodological quality of evaluations implemented to measure their effects [26-29]. Therefore, this systematic review aims to: Firstly, identify evaluations of suicide prevention interventions targeting Indigenous peoples in Australia, New Zealand, Canada and the United States published in the scientific and grey literature; secondly, critique their methodological quality using a standardised assessment tool; and thirdly, describe their key characteristics. The PRISMA guidelines [30] for reporting of systematic reviews were followed in carrying out this study and preparing the manuscript.

## Methods

### Search strategy

**Figure 1:**
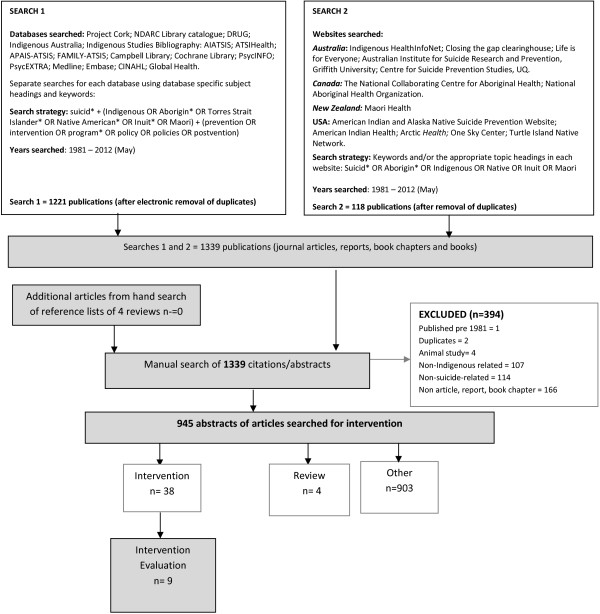
Flowchart of search strategy.

Figure 1 summaries the databases searched, the search terms used, the exclusion criteria, and classification of included studies. Consistent with methods detailed in Cochrane Guidelines for systematic reviews [31], and used in previous systematic reviews [32,33], the search strategy comprised three steps. First, consultation with a qualified librarian identified 17 relevant electronic databases to search (Figure 1, Search 1): Project Cork; NDARC Library catalogue; DRUG; Indigenous Australia; Indigenous Studies Bibliography: AIATSIS; ATSIHealth; APAIS-ATSIS; FAMILY-ATSIS; Campbell Library; Cochrane Library; PsycINFO; PsycEXTRA; Medline; Embase; CINAHL; Global Health. The terms suicid* and Aborigin* OR Indigenous OR Torres Strait Islander* OR Native American* OR Inuit OR Maori were searched using keywords and subject headings specific to each database. All subject headings were exploded so that narrower terms were included. The combined searches of the 17 databases (excluding duplicates) identified 1221 references that were imported into Endnote. Second, to maximise search coverage of the grey literature, 13 websites and clearinghouses related to Indigenous peoples of Australia, New Zealand, Canada and/or the USA were also searched (Figure 1, Search 2). 118 studies not identified in the electronic database search were identified. Third, reference lists of reviews of suicide prevention interventions targeting Indigenous peoples of Australia, Unites States, Canada and/or New Zealand [26-29], identified by the electronic database search, were hand-searched for relevant studies not yet identified. No additional studies were identified. In total, 1339 references were identified for classification.

### Classification of studies

The titles and abstracts of the 1339 identified references were classified in a three step process.

#### Step 1: Identification of studies for exclusion

Papers were excluded if they: (a) were duplicates (n=2); (b) did not focus on suicide, or if the outcomes or predictor variables did not include or specifically relate to suicide (n=114); (c) did not focus on Indigenous people in Australia, New Zealand, United States or Canada (n=107); (d) were not journal articles, reports or book chapters (n=166); (e) were an animal study (n=4); or (f) were published pre 1981 (n=1). Step 1 excluded 394 papers, leaving 945 references.

#### Step 2: Classification of studies

The abstracts of remaining studies (*n* = 945) were examined by the first author (AC) to identify studies that were (i) *Intervention,* defined as studies on suicide prevention, early intervention or postvention program, service or policy targeting Indigenous people (n=38); or (ii) Reviews, defined as literature reviews of suicide prevention interventions, (n=4). Abstracts of studies that were not intervention or review (i.e. descriptive, analytical or measurement research unrelated to an intervention) were classified as other (n=903). Twenty percent (*n* = 180) of studies were re-classified by a research assistant blinded to the results of the initial classification, for cross-checking. Agreement between classifiers was substantial (kappa=0.68). Sufficient agreement between co-authors deemed crosschecking more than 20% of article classifications unnecessary. The articles excluded in Step 1 were not cross-checked because they were not relevant to the review. The 38 intervention studies identified in Step 2 were retained for further examination.

#### Step 3: Identification of intervention evaluations

The journal article of intervention-related studies identified in Step 2 (n=38) were obtained and examined to identify intervention evaluations – studies evaluating that a suicide prevention, early intervention or postvention program, service or policy. Ten intervention evaluations were identified [34-43]. The remaining 28 publications were excluded on the basis they described the development or implementation of an intervention but did not evaluate its effectiveness. Of the 10 intervention evaluations, two reported outcomes for the same intervention [35,36], leaving 9 intervention evaluations for methodological review.

### Data extraction from intervention studies

Criteria for data extraction from studies were adapted from the Cochrane Collaboration Handbook for Systematic Reviews of Health Promotion and Public Health Interventions [31]. The criteria, shown in Table 1, relate to the intervention/s, the sample (including eligibility, size, age range and percent male), study design, the outcomes measured, follow-up period, effects, and the cost calculations performed.

### Methodological critique of intervention studies

Methodological quality was assessed using the Dictionary for the Effective Public Health Practice Project Quality Assessment Tool for Quantitative Studies [30]. Sections A to F (A. selection bias; B. allocation bias; C. confounders; D. blinding; E. data collection methods; and F. withdrawal and drop-outs) were coded weak, moderate or strong, consistent with the component rating scale of the Dictionary [31]. For Sections G (analysis) and H (intervention integrity), descriptive information was recorded in line with the Dictionary's recommendations.

**Table 1:** Characteristics of evaluations of suicide prevention interventions

**1st author, year, publication type**	**Country, location**	**Intervention type**	**Intervention component/s (number of sessions)**	**Target group (n), age, % male**	**Design**	**Data collection methods**	**Outcomes**	**Effects**	**Follow-up months**	**Cost**
La Fromboise, 1995, journal article [41]	US, rural New Mexico	Education	Culturally tailored school-based life skills curriculum, including manual and teacher raining. (3/week × 30 weeks)	Native Americans (n=128): age range= 14–19 years, mean age=15.9 years and 36% male.	Pre-post, with two control groups	Self-report survey Observational methods	Suicide vulnerability: hopelessness, depression and self-efficacy	Intervention group less hopelessness (P<0.05); less suicidal (P<0.07); not less depressed.	8 months	NR
Berman, 1999, journal article [42]	US, rural Alaska	Community prevention	Alcohol restrictions	Experimental: 29,000 Control: 21,000	Interrupted time series with control group	Routinely collected population level data	Death rates from accidents, suicides and homicides	Significant reductions (*P*<0.05) in homicide for high level restrictions, and in suicide for low level restrictions. 21% reduction in overall injury deaths.	1-13yrs	NR
Tsey, 2000, journal article [43]	Australia, remote Qld	Community prevention	Four stage empowerment program (1 × 4hr session per week for 10 weeks per each stage)	Aboriginal community members (n=31): age range= 20–50 years, median age=early 40 years, and 10% male	Pre-post, no control	Self-report survey Participant observation Narrative interviews	Individual & community levels of empowerment	NR	10, 20, 30, and 40 weeks	
May, 2005, journal article [39]	US, rural New Mexico	Community prevention	Train youth as natural helpers	Native Americans (n=800): age range = 10–19 and 20–24 years.	Interrupted time series, no control	Self-report by health professionals and police and medical records	Suicide attempts, gestures & completions	Significant reductions (*P*<0.05) in rates of suicidal gestures and attempts. No change in suicidal completions.	13yrs	NR
			Drug and suicide education							
			Family outreach post-suicide							
			Suicide-risk screening							
			Community cultural events							
			Reorientation of mental health services							
Deane, 2006, journal article [35,36]	Australia, regional NSW	Gatekeeper training	Suicide awareness and skills Gatekeeper training (8 × 1 day workshops)	Aboriginal Australian community members (n=48): age range= 19–55 years, mean age=36 years and 9% male.	Pre-post, no control	Self-report survey Interviews	Knowledge	Significant increases (*P*<0.05) pre-post training in knowledge, intentions, confidence. Non-significant changes post training to 2 years follow-up.	2yrs	NR
							Confidence			
							Intentions			
Haggarty, 2006, journal article [34]	Canada, rural	Education	Multi-media education (1 × 30 minute session)	Healthcare providers, teachers, students and elders (n=24)	Pre-post, no control	Self-report survey	Knowledge	Significant increases (*P*<0.05) in knowledge	NR	NR
Westerman, 2007, grey report [38]	Australia, rural and remote Western Australia	Gatekeeper training	Training and information workshops	Aboriginal youth and community members (n=769): age range =15-25 years.	Pre-post, no control	Self-report survey Interviews	Knowledge Confidence Intentions	Significant improvements (*P*<.05) in knowledge and confidence in how to identify individuals at risk of suicide.	NR	NR
Muehlenkamp, 2009, journal article [37]	US, Native American	Gatekeeper training	Gatekeeper training, education workshops, social activities, individual counselling and education seminars, student support team, social networking, spiritual ceremonies	Native American college students (n=90)	Pre-post, no control	Self-report survey	Knowledge Attitudes	Reported improvements in problem solving ability, and marginal improvements in communication skills and knowledge	NR	NR
							Skills			
Allen, 2009, journal article [40]	US, remote Alaska	Community prevention	Community module: 26 prevention activities (7 targeting community) in 32 sessions. Additional activities: increased alcohol control, suicide crisis response team & prayer walks (32 sessions over 12 months)	Alaskan Indigenous youth (n=61): age range=12-17 years, mean age= 14 years, and 30% male.	Pre-post, no control	Self-report survey	Community readiness	Significant (*P*<0.05) increase in number of protective behaviours in youth.	NR	NR
							Youth & adult protective behaviours			
				Adults of youth (n=47): mean age=48 years and 42% male.						
				Community informants (n=5)						

Note. NR=Not reported.

## Results

Table 1 summarises the characteristics of intervention evaluations.

### Indigenous population and sample

Five intervention evaluations targeted Native Americans [37,39-42]; three targeted Aboriginal Australians [35,36,38,43] and one First Nation Canadians (Inuit) [34]. No interventions targeted the Maori of New Zealand. The sample population reported by studies included both Indigenous young people and general community members [38,40]; young people only [37,41]; defined young and/or adult sub-populations within communities [35, 39 43]; and whole communities [42]. Six studies reported the age of participants, ranging from 10 to 55 years of age [35,38-41,43]. Five studies reported the percentage of male participants: 9% [35]; ~10% [43]; 26% [34]; 36% [41]; and 30% (youth) and 42% (adults) [40].

### Intervention strategies

The main intervention strategies employed included: community prevention initiatives [39,40,42,43]; gatekeeper training [35-38]; and education programs [34,41].

#### Community prevention

Four intervention studies employed community prevention strategies targeting Indigenous groups and communities at high risk of suicide [39,40,42,43]. Two studies employed one main strategy: one employed alcohol restrictions in multiple Native Alaskan communities [42], and the other a structured Aboriginal-specific empowerment program in Australia [43]. The other two community prevention studies combined multiple strategies in a community prevention intervention [39,40]. ‘The Adolescent Suicide Prevention Project’ integrated multiple strategies within a public health framework, including: training of youth as natural helpers; drug and suicide education; family outreach post-suicide; suicide-risk screening; community social and cultural events; and the reorientation and expansion of mental health services [39]. ‘The Elluam Tungiinun’ prevention program developed a toolbox of community prevention modules for delivery within a cultural framework of community development [40]. Modules were complemented with other strategies, including, alcohol controls, prayer walks and a suicide-crisis response team [40].

#### Gatekeeper training

Gatekeeper training involves teaching specific groups of people in the community how to identify and support individuals at high risk of suicide. Three studies evaluated the effectiveness of gatekeeper training [35-38], all of which reported that gatekeeper training programs were developed in consultation with targeted Indigenous groups and communities [35-38]. Two studies evaluated the effectiveness of gatekeeper training only [35,36,38], and one evaluated gatekeeper training combined with other strategies, including individual counselling, education and support, and group-based social and cultural activities [37]. Two gatekeeper training interventions appeared to be delivered in a defined number of sessions over a short time period [35,37], while one was delivered in three stages over 18 months, ‘to enable participants and their communities to develop their knowledge and skills over time’ [38].

#### Education

Two studies employed an education intervention as their main strategy: one integrated culturally tailored life skills training (e.g. communication and problem solving) into the high school curriculum for delivery to Native American teenagers [41], and the other delivered a one-off multi-media education session to interested community members [34]. Two studies employing community prevention as a main strategy included an educational component [39,43]. Of the four studies employing an educational strategy (as a main or minor component), two reported developing a new [34] or adapting [43] an existing education resource for delivery, and one reported training intervention deliverers [41].

### Methodological adequacy

Table 2 summarises the methodological adequacy of intervention evaluations.

**Table 2:** Methodological adequacy of evaluations of suicide prevention interventions

**1st author, year**	**Selection bias (A)**	**Allocation Bias (B)**	**Confounders (C)**	**Blinding (|D)**	**Data collection Methods (E)**	**Withdrawal & drop-outs (F)**	**Analysis (G)**	**Intervention integrity (H)**
La Fromboise 1995 [41]	Weak	Moderate	Moderate	N/A	Moderate	Moderate	-Citation for formula used in the analysis	-No consent rate reported, 76% follow-up rate.
							-High response rate	-Number of intervention sessions received by participants not reported.
								- Manual used with teacher training
								-Random observations of intervention delivery by intervention co-ordinator
Berman, 1999 [42]	Strong	Strong	Strong	N/A	Strong	N/A	-Citations to justify analysis but no citations for analysis method	-Communities level of exposure to alcohol control reported and considered in analysis
Tsey, 2000 [43]	Weak	Weak	Weak	N/A	Weak	Moderate	Citation to justify theory but not analysis	-No consent rate reported and follow-up rate only partially reported
								-Adaptation of existing Aboriginal-specific program,
								-Components of each stage described
May, 2005 [39]	Moderate	Weak	Weak	N/A	Strong	N/A	-No citation for formula used in the analysis	- Number and type of prevention activities recorded but reported elsewhere
								- Staff growth for program delivery reported
Deane, 2006 [35,36]	Moderate	Weak	Weak	N/A	Moderate	Strong	- Citations to justify analysis but no citations for analysis method	-93% consent rate and 91% and 100% follow-up reported.
								-Manual for tailored delivery, dependent on group's needs
							-High response rate reported	
Haggarty, 2006 [34]	Weak	Weak	Weak	N/A	Weak	Moderate	-No citation for analysis method	- 79% follow-up
								- length of time of participant’s exposed to multi-media resource recorded
Westerman, 2007 [38]	Weak	Weak	Weak	N/A	Moderate	Moderate	No description of analysis or citation.	-Consent rate not reported and 77% follow-up
								-Intervention delivered by Indigenous Psychological services
Muehlenkamp, 2009 [37]	Weak	Weak	Weak	N/A	Moderate	Weak	No citation for analysis method	-No consent rate reported and follow-up rate difficult to determine
								-Some report of intervention exposure
								-Adaptation of existing intervention
Allen, 2009 [40]	Moderate	Weak	Strong	N/A	Moderate	Strong	-Citation for formula used in the analysis.	-61% consent rate reported for individual program component
							-Low to moderate response rates	Intervention toolkit for tailoring to local needs
								-Intervention exposure (number and type of activities) measured and considered in analyses

Seven studies used a pre-post study design [34,35,37,38,40,41,43]; six did not employ a control group [34,35,37,38,40,43], making it difficult to attribute outcomes reported to the intervention. Two studies employed a time series design, one with [42] and the other without a control group [39]. No study employed randomisation, increasing the risk of selection bias. Seven studies reported using a previously tested or validated measure and provided a citation to justify its selection [35-42]. Of the six studies in which it was appropriate to report consent rates, four did not [37,38,41,43] and two reported consent rates of 93% [35] and 61% [40] respectively. Follow-up rates were fully reported by three [38,40,41] of the six relevant studies and ranged from 76% [41] to 100% [40].

Six studies reported tailoring the intervention prior to its implementation to improve its acceptability to Indigenous peoples. Methods of tailoring included Indigenous community input and/or feedback [35-41,43], piloting intervention materials [38,40,41], integration of Indigenous culture into intervention content [35,38,40,41,43] and researching suicide in the target population [38]. The intervention study evaluating the impact of alcohol restrictions reported that the restrictions were initiated by Indigenous communities [42].

Methods to optimise consistency in intervention delivery were described by five studies and included training intervention deliverers [41], intervention manuals or packages [35,38,40,41,43] and/or self-report or observation [41,43]. One intervention was developed by an Indigenous-specific psychological service [38] and another by Indigenous survivors of the stolen generation in Australia [43].

Seven studies recorded participant attendance at intervention activities to measure their level of exposure to the intervention [34,38-41,43], one of which also reviewed participants’ clinical records [39]. The study evaluating the impact of alcohol restrictions measured the level of, and period of exposure to, restrictions in each intervention community [42].

### Data collection methods and outcomes

Seven studies used self-report measures only: three used self-complete surveys only [34,37,40], two used self-complete surveys and interviews [35,38], one self-complete survey and observation [41], and one self-complete surveys, interviews, and observation [43]. Two studies used routinely collected community level data [39,42], one of which complemented this with self-report interviews [39]. Only two studies measured suicide specific outcomes, including suicide attempts [39,42], gestures [39] and completions [39,42]. Four studies measured changes in knowledge, confidence and/or intentions to identify and assist individuals at risk of suicide [34,35,37,38]. One study measured psychological risk factors for suicide, including depression, vulnerability, and feelings of hopelessness [41]. One study reported targeting the whole community but only measured individual level outcomes [40], while another reported positive changes among intervention participants but did not report the measures used [43].

### Effectiveness of interventions

Heterogeneity in study methodology and outcomes reported limited formal meta-analysis. Notwithstanding this, some observations are made. Gatekeeper training resulted in significant (*P*<0.05) short-term increases in participants’ knowledge and confidence in how to identify individuals at risk of suicide, and their intention to help those at risk of suicide [35,37,38]. For education interventions, students receiving a culturally tailored suicide prevention intervention were less suicidal (*P*<0.07) and showed significantly (*P*<0.05) less feelings of hopelessness than those that did not [41], while a one-off multi-media intervention significantly improved (*P*<0.04) participants’ knowledge of risk behaviours at post-test [34]. Two of the four community prevention interventions reported significant (*P*<0.05) reductions in rates of suicide [42] or suicidal behaviours (life threatening self-inflicted injury) [39]. For the other two community prevention interventions, one reported significant (*P*<0.05) increases in the number of protective behaviours among youth exposed to intervention activities [40], while the other reported subjective improvements in protective factors for suicide among intervention participants [43].

## Discussion

Consistent with previous reviews [25-28], few published evaluations of Indigenous-specific suicide interventions were identified in the peer review and grey literature, and the methodological quality of studies was less than optimal. Evaluating Indigenous health interventions is complex and challenging [44]. Indigenous communities and researchers may have too many competing priorities which conflict with the time and effort required to rigorously evaluate suicide prevention interventions in Indigenous communities. They may also lack the necessary skills and expertise. Indigenous communities are unlikely to have skills and expertise in intervention evaluation if there is limited opportunity for them to work with researchers experienced in this field [45]. Evaluations of Indigenous suicide interventions that are scientifically rigorous, engage Indigenous peoples as equal partners in the research process, and build Indigenous research capacity are likely to be expensive. Funding agencies may not be able to afford or be willing to fund substantial budgets for evaluations of suicide prevention interventions in Indigenous communities. The predominance of descriptive research in the Indigenous health research field is an indication of the difficulties researchers and Indigenous communities face addressing the complexities and challenges associated with undertaking Indigenous intervention research [44].

### Methodological quality

The methodological quality of studies varied considerably and none had consistently strong methodology across the majority of applied criteria. Weak ratings were commonly recorded for selection bias, allocation bias and confounding. Few study designs employed a control group and none employed randomisation. These findings are consistent with previous reviews of Indigenous intervention research [46,47], and provide an opportunity for researchers to improve the quality of evaluations of Indigenous suicide prevention interventions through the application of more rigorous study designs. Encouragingly, data collection methods were generally moderate to strong: seven of the nine studies used a previously tested or validated measure. Also encouraging was the finding that most interventions were tailored to optimise their acceptability, and standardised to reinforce their delivery, to Indigenous peoples.

### Strengths and limitations of interventions

Interventions typically employed suicide prevention strategies with some evidence for their effectiveness. However, strategies with the strongest evidence were typically not employed. For example, only one intervention implemented suicide-risk screening [39], despite evidence from non-Indigenous populations that routine screening of individuals at high risk of suicide (e.g. young people) is effective for detecting those at risk of suicidal behaviour and, in some instances, has led to reductions in suicide deaths [14]. Researchers may be unable to implement some evidence-based strategies in Indigenous communities: a strategy may be too difficult to implement (e.g. it may require extensive tailoring to be acceptable and feasible) or Indigenous people may find it unacceptable.

Appropriately, gatekeeper training employed educational strategies to improve gatekeepers’ intent to respond to individuals at risk of suicide [25]. Nevertheless, as with evaluations of gatekeeper training interventions in non-Indigenous communities [14,25], future evaluations of gatekeeper training in Indigenous communities would be strengthened by the measurement of intermediate outcomes, such as referral and treatment rates of individuals identified at risk of suicide [14].

Consistent with findings from studies in non-Indigenous populations [14], the school-based suicide prevention strategy in this review reduced young peoples’ feelings of depression and hopelessness [40], but its effect on their suicidal behaviours was not measured. Although school-based programs offer great potential to reach large numbers of young people [32], there is no evidence that they reduce suicidal behaviour in the absence of other strategies [14]. Furthermore, it is highly questionable whether or not school-based programs are likely to reach Indigenous young people most at risk of suicide, given that high risk young people typically attend school irregularly or not at all [29].

No study considered intervention costs. Economic analysis of suicide preventive interventions is important for understanding resources used and the potential cost-effectiveness of strategies designed to avert suicide deaths and suicidal behaviours, and subsequent economic and social savings [48]. Although the economic costs of suicide in Indigenous populations has not been quantified, the profound negative impact of suicide on the social and emotional wellbeing and psychological functioning of affected Indigenous individuals, families and communities [7,15] strongly suggests they are likely to be high and accumulate over a lifetime.

### Recommendations and future directions

Overall, the results of this review suggest there is insufficient evidence from published evaluations as to which intervention strategies are most effective for preventing suicide among Indigenous peoples in Australia, New Zealand, Canada and the United States. A number of clear recommendations can be posited. First, effective partnerships between government and research agencies, health-care providers and Indigenous health-care services are required to increase the likelihood that methodologically rigorous evaluations of suicide prevention programs in Indigenous communities are undertaken. These evaluations should be designed with researchers with the relevant skills, and need not be expensive if they occur simultaneously with the development and implementation of a suicide prevention policy or program. Second, given the lack of Indigenous-specific evidence, tailoring evidence-based suicide prevention strategies to the needs and preferences of Indigenous communities [35,38,49], and evaluating cultural specific suicide prevention programs, is likely to be required [2,7]. Both processes will require strong collaborative partnerships between researchers and Indigenous communities to enable reciprocal exchange of knowledge, practices and ideas. Third, community-wide interventions co-ordinating a series of strategies targeting common risk factors for suicide (i.e. mental health disorders, alcohol abuse and a prior history of self-harm) should be designed and implemented in collaboration with Indigenous communities and their impact and economic costs rigorously evaluated. The results of such an evaluation would improve the effectiveness of future policies and programs designed to reduce rates of Indigenous suicide. Fourth, alongside intervention research, quality measures research is needed to ensure that Indigenous suicide data is accurate and reflect cultural definitions of health and wellbeing from the perspective of Indigenous peoples.

#### Potential limitations of the review

Although a rigorous and thorough search strategy was used, there is the possibility that the review did not locate all relevant studies. Relevant intervention evaluations may have been misclassified. However, a high level of agreement between blinded coders suggests not. Since evaluations with statistically significant findings are more likely to be published, it is possible that the published evaluations reviewed over-estimate the true effectiveness of suicide prevention intervention targeting Indigenous peoples [50].

## Conclusions

The urgent need to reduce the disproportionately high rates of suicide in Indigenous peoples of Australia, New Zealand, Canada and the United States has been widely acknowledged. In order for this to occur, an increase in the number of evaluations of preventive interventions targeting reductions in Indigenous suicide using methodologically rigorous study designs across geographically and culturally diverse Indigenous population groups is required. While evaluations of suicide prevention interventions in discrete Indigenous communities using non-experimental designs may be easier and cheaper to implement, they are unlikely to provide strong evidence applicable to other Indigenous populations. Without this evidence there is an increased likelihood that ineffective interventions will be implemented to prevent suicide in Indigenous peoples of Australia, New Zealand, Canada and the United States, reducing the likelihood of achieving significant reductions in rates of suicide in these populations.

## Competing interests

The authors declare that they have no competing interests.

## Authors’ contributions

AC, CD and KT contributed to the search strategy. AC took the lead role in reviewing the quality of intervention publications and drafted the paper. CD and KT revised the methods and edited the draft paper. All authors read and approved the final manuscript.

## Pre-publication history

The pre-publication history for this paper can be accessed here:

http://www.biomedcentral.com/1471-2458/13/463/prepub
